# Preparation of polyaniline/PbS core-shell nano/microcomposite and its application for photocatalytic H_2_ electrogeneration from H_2_O

**DOI:** 10.1038/s41598-018-19326-w

**Published:** 2018-01-18

**Authors:** Mohamed Rabia, H. S. H. Mohamed, Mohamed Shaban, S. Taha

**Affiliations:** 1 Nanophotonics and Applications Lab, Physics Department, Faculty of Science, Beni-Suef University, Beni-Suef, 62514 Egypt; 2 Polymer Research Laboratory, Chemistry Department, Faculty of Science, Beni-Suef University, Beni-Suef, 62514 Egypt; 3 Physics Department, Faculty of Science, Fayoum University, EL-Fayoum, Egypt

## Abstract

Lead sulfide (PbS) and polyaniline (PANI) nano/microparticles were prepared. Then, PANI/PbS core-shell nano/microcomposites (I, II, and III) were prepared by oxidative polymerization of different aniline concentrations (0.01, 0.03, and 0.05 M), respectively, in the presence of 0.05 M PbS. FT-IR, XRD, SEM, HR-TEM, and UV-Vis analyses were carried out to characterize the samples. From the FT-IR data, there are redshifts in PbS and PANI nano/microparticles bands in comparison with PANI/PbS nano/microcomposites. The average crystallite sizes of PANI/PbS core-shell nano/microcomposites (I, II, and III) from XRD analyses were 46.5, 55, and 42.16 nm, respectively. From the optical analyses, nano/microcomposite (II) has the optimum optical properties with two band gaps values of 1.41 and 2.79 eV. Then, the nano/microcomposite (II) membrane electrode supported on ITO glass was prepared and applied on the photoelectrochemical (PEC) H_2_ generation from H_2_O. The characteristics current-voltage and current-time behaviors were measured at different wavelengths from 390 to 636 nm. Also, the incident photon-to-current conversion efficiency (IPCE) under monochromatic illumination condition was calculated. The optimum values for IPCE were 36.5 and 35.2% at 390 and 405 nm, respectively. Finally, a simple mechanism for PEC H_2_ generation from H_2_O using the nano/microcomposite (II) membrane electrode was mentioned.

## Introduction

The economic model and the present global society are the most critical challenges that can be confronted the world in 21^st^ for the provision of clean and renewable energy to satisfy the increasing human demands. Renewable and cleaning energy can be offered by sunlight^1^. In this context, hydrogen (H_2_) is considered as the cleanest and environmentally friendly energy; it has an ideal fuel for the future. Photoelectrochemical (PEC) H_2_ generation can be occurred by water splitting with semiconductor materials. This technique is the most straightforward conversion because only water and sunlight can produce H_2_ and the combustion process in hydrogen fuel cells leads to electricity with water as the only by-product^2,3^.

In recent years, H_2_ has become a hot academic topic since Fujishima and Honda were the first who worked in photocatalytic water splitting using a TiO_2_ electrode^4^. These advances have motivated the search for a growing list of applications of the functional materials^5–7^. Among these materials, metal sulfide semiconductors **(**SCs) have attracted broad interest in photocatalysis and solar cell applications because of their tunable band gap and unique optical properties^8–11^. In particular, lead sulfide (PbS) is one of the metal sulfides which has promising photosensitive properties and can be prepared as n or p-type for solar energy applications due to the variety of band gaps that affected by the particle sizes^12,13^. Moreover, the absorption capacity of PbS nanomaterials in many regions of the light and can reach to the infrared region in wavelengths ~800–1700 nm, with a percent of about 40% of the solar radiation on the earth’s surface. So PbS nanoparticles could improve light absorption very much^14^. The metals sulfides SCs can be prepared by several techniques such as thermal evaporation, spray deposition, and chemical bath deposition (CBD). CBD is the most widely used method due to its simplicity and low-cost effectiveness, and it can occur at low temperature^15^.

In addition, there are some studies depended on PbS materials in photocatalytic water splitting for solar hydrogen generation. Chih-Hsiung *et al*. showed decoration of PbS nanoparticles on Al-doped ZnO nanorod array thin film as a photoelectrode for solar water splitting leading a maximum photocurrent density of 1.65 mW cm^−2^ ^16^. The study carried out from solution contained 0.25 M Na_2_S and 0.35 M Na_2_SO_3_ at pH = 13 using 350 W Xe lamp. Hernandez-Borja *et al*. prepared PbS/CdS by CBD process and applied it as photoelectrode with an efficiency of 1.63% for solar energy applications^17^. Cui *et al*. synthesized PbS intercalated K_2_La_2_Ti_3_O_10_, by ion-exchange reaction under the microwave irradiation. But the amount of H_2_ generated was small of about 127 mmol/g after 3 h irradiation of ultraviolet light^18^.

Moreover, the enormous progress in the synthesis of the composite core-shell structures has significantly advanced research ability to tune their optical, electrical, mechanical, and chemical properties for using them in new photo/electro-applications^19^. On the other hand, a significant interest among researchers has been aroused to conducting polymers due to their curious electronic, high conductivity, magnetic and optical properties, high stability, simple preparations, and good environmental compatibility^20–22^. Because of these useful properties, the photoactivity can be enhanced by using PANI as a photosensitizer to modify the photocatalysts^23^. The presence of conjugated π electrons along the backbone of polymer like PANI provides the ability to support positive as well as negative charge carriers. Exciting and unique physical properties are expected when the delocalized carrier sinter acts with the semiconductor quantum dots with high mobility along the chain. There were variously modified photocatalysts based on PANI such as PANI/TiO_2_^24^, PANI/BiVO_4_^23^, PANI/SnO_2_^25^, and PANI/MoO_3_^26^. Wang *et al*. have designed and synthesized monodisperse PANI@CdS core-shell nanospheres to probe the mechanisms of photocorrosion inhibition and photocatalytic H_2_ production^27^. Zhang *et al*. revealed that the exciting enhanced visible light photocatalytic activity and excellent anti-photocorrosion performance of CdS photocatalysts were obtained after hybridized by monolayer PANI^28^.

The efficiency of PEC H_2_ production using these composites still limited and the designed nanocomposites efficiency need more enhancement^27,29^. Moreover, many studies used high-cost techniques and depended on chemical substances such as CH_3_COOH, HCL, HNO_3_, and H_2_SO_4_ as a source for H_2_^30^. So the critical issues for H_2_ generation process are to be commercially available, reducing the synthesis and applications cost, and enhance the stability of the used photocatalysts^31–34^.

In this work, the synthesis of PbS and PANI nano/microparticles, and PANI/PbS core-shell nano/microcomposites (I, II, and III) using simple and low-cost techniques was carried out. Moreover, the structural, morphological, and optical properties of the fabricated nanostructures were investigated using different characterization techniques. Also, the effect of aniline concentrations on the properties of the fabricated nano/microcomposites was studied. Moreover, PEC water splitting properties and efficiencies such as I-V and I-T responses were studied on PANI/PbS/ITO core-shell nano/microcomposite (II) membrane electrode. Also, IPCE under monochromatic illumination conditions was addressed.

## Results and Discussion

### Nano/microparticles characterization

**Figure 1:**
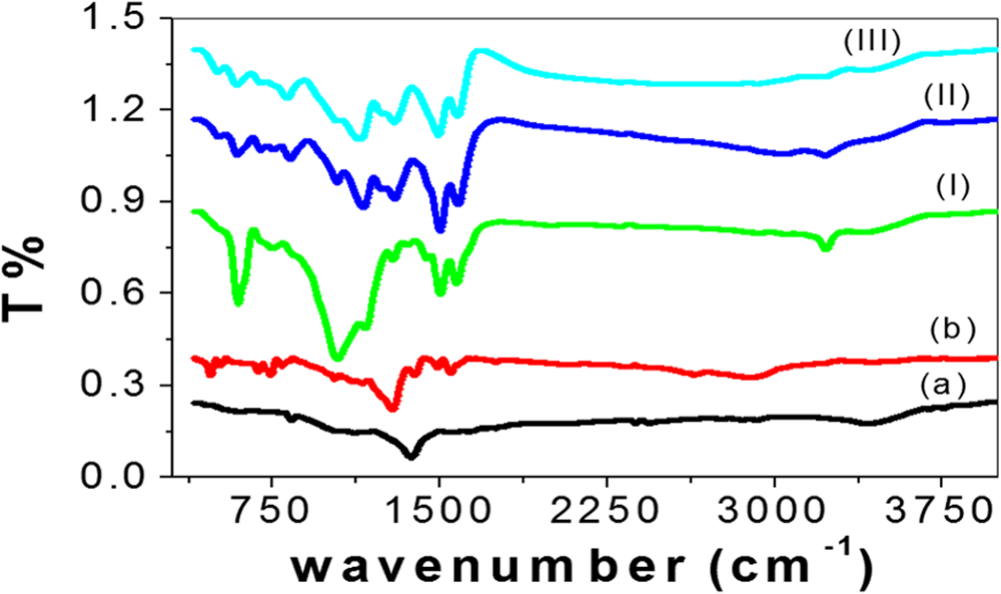
FTIR analyses of (**a**) PbS and (**b**) PANI nano/microparticles, and PANI/PbS core-shell nano/microcomposites (I, II, and III).

It is known that the surface structures of the PANI, PbS, and PANI/PbS core-shell nano/micromaterials influence their optical properties, for this reason, it was imperative to investigate their structures and morphologies. The structural analyses of all nano/microparticles were examined, in which the FT-IR spectra are shown in Fig. 1. In addition, the data of these nano/microparticles are mentioned in Table 1. From the figure and table, there are redshifts of bands of PbS (a) and PANI (b) nano/microparticles in comparison with that observed for the PANI/PbS nano/microcomposites (I, II, and III). This appears in the frequencies of the heteropolar diatomic molecule in PbS and N–H stretching vibrations of amino groups in PANI. These shifting may result from the increased energy of interaction between PbS and PANI levels. While a little blue-shift in bands in all core-shell nano/microcomposites (I, II, III) with increasing the PANI ratio in the composite from I to III. These shifts are a result of the interaction between the constituents of the formed nano/microcomposites^35^.

**Table 1:** The FTIR analyses of the PbS, PANI, and PANI/PbS nano/microcomposites (I, II, and III).

Band position (cm^−1^)					Assignment
PANI/PbS composite I	PANI/PbS composite II	PANI/PbS composite III	PbS	PANI	
3229	3226	3221	—	3401	N–H stretching vibrations of amino groups in the nano/microcomposite^58^
2925	2922	2920	—	2918	Vibration of C-H aromatic ring
1501	1500	1492	—	1530	The coordinated water molecule and C = C stretching vibrations of quinoid ring^59^
1289	1295	1295	1357	—	The frequencies of heteropolar diatomic molecules of lead sulfide^60^
1301	1301	1300	—	1301	C = C vibration of benzoid rings
1165	1155	1137	—	1138	C–N stretching vibrations
1041	1039	1040	1061	—	The frequencies of heteropolar diatomic molecules of lead sulide^60^
747	827	814	—	798	The aromatic C−H out-plane bending
596	592	590	—	587	Para disubstituted aromatic rings^61^

**Figure 2:**
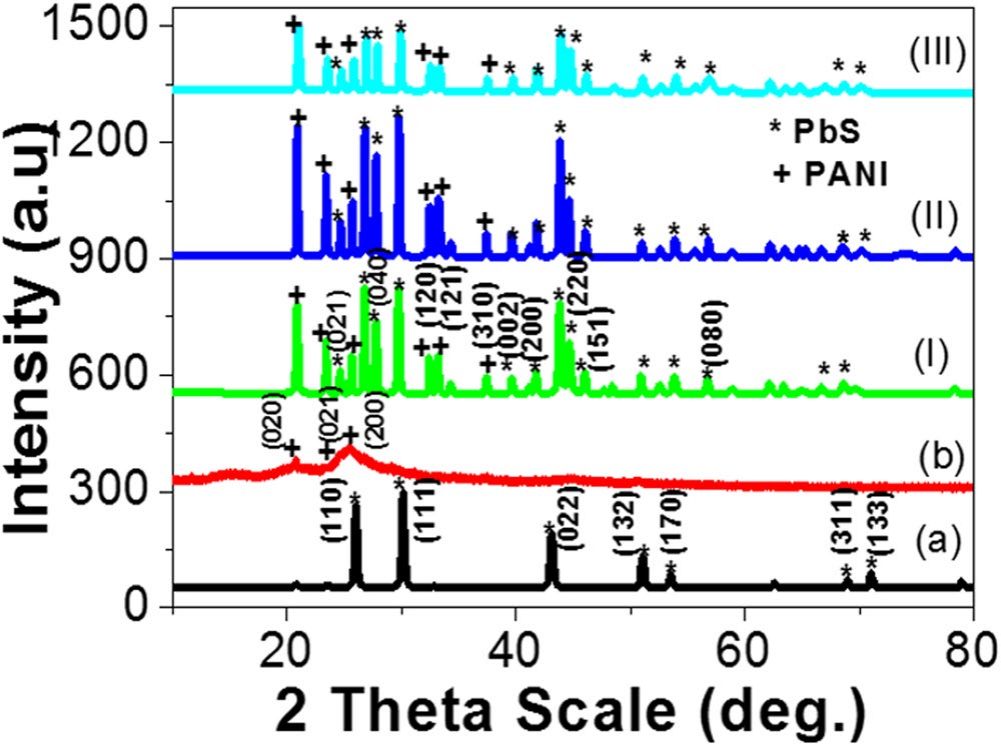
X-ray analyses of (**a**) PbS and (**b**) PANI nano/microparticles, and PANI/PbS core-shell nano/microcomposites (I, II, and III).

The X-ray diffractometers at room temperature for PbS and PANI nano/microparticles, and PANI/PbS core-shell nano/microcomposites (I, II, and III) are shown in Fig. 2. The X-ray analysis of PbS particles is shown in Fig. 2(a). There are seven distinct crystalline peaks appeared centered at 2θ = 25.98°, 30.17°, 43.12°, 51.14°, 53.70°, 68.82°, and 71.01° corresponding to (110), (111), (022), (132), (170), (311), and (133), respectively. The average crystallite size of the PbS particles is determined using the full width at half maximum (W) data. The determination process takes place using Scherrer’s formula^35^ D = 0.9λ/W cosθ; where W is in radians, θ is the Bragg’s angle, λ is the X-ray wavelength (CuKα = 0.15405 nm). From the calculation process data, the average crystallite size of the PbS particles is ~36.3 nm.

The XRD patterns of PANI particles is illustrated in Fig. 2(b). The XRD spectrum of PANI clearly indicated the preparation of PANI crystallites with crystalline domains. Three distinct crystalline peaks appeared centered at 2θ = 15.18°, 21.12°, and 25.49°, which corresponding to (020), (021), and (200) crystal planes, respectively, of PANI in its emeraldine salt form^36^. The characteristic peaks at 2θ = 15.18° and 25.49° are ascribed to the perpendicular and parallel periodicity of the polymer chain, respectively^37,38^. The average size of the PANI crystallites is determined from the full width at half maximum (W) in radians using Scherrer’s formula; D = 0.9λ/W cos θ; where λ is the X-ray wavelength (CuKα = 0.15405 nm)^35^. The calculated value of the average crystallite size of the PANI is ~100 nm.

The XRD patterns of PANI/PbS core-shell nano/microcomposites (I, II, and III) appear in Fig. 2 (I–III). From the figure, the formation of crystalline Six distinct crystalline peaks appeared centered at 2θ = 15.18°, 21.12°, 25.49°, 32.4°, 33.1°, and 41,8° corresponding to (020), (021), (200), (120), (121), and (310), respectively, for crystal planes of PANI in its emeraldine salt form^39^. In addition, the two characteristic peaks at 2θ = 15.18°, 23.2° are ascribed to the perpendicular and parallel periodicity of the polymer chain, respectively^40^. Moreover, there are 14 distinct crystalline peaks for PbS core nanoparticles appeared centered at 2θ = 24.4°, 25.98°, 27.7°, 30.17°, 39.6°, 41.8°, 43.12°, 44.3°, 45.9°, 51.14°, 53.70°, 68.82°, and 71.01° corresponding to (021), (110), (200), (111), (002), (200), (022), (220), (151), (132), (170), (311), and (133). The average crystallite sizes of the PANI/PbS core-shell nano/microcomposites (I, II, and III) are determined from the full width at half maximum (W) data. From the calculation process data, the average crystal sizes of the PANI/PbS core-shell nano/microcomposites (I, II, and III) are 46.5, 55, and 42.16 nm, respectively. The increasing in size with aniline concentrations from 0.01 M (composite I) to 0.03 M (composite II) is due to increasing of the nucleation sites that cause more coalescence of the deposited nano/microcomposite^41^. But with further increasing of the aniline concentration to 0.05 M (composite III), the particles sizes decrease due to the reduction of the Vander Waals force interactions that exist between the crystals because of disorder the particles deposition during the formation of core-shell composite^42^.

**Figure 3:**
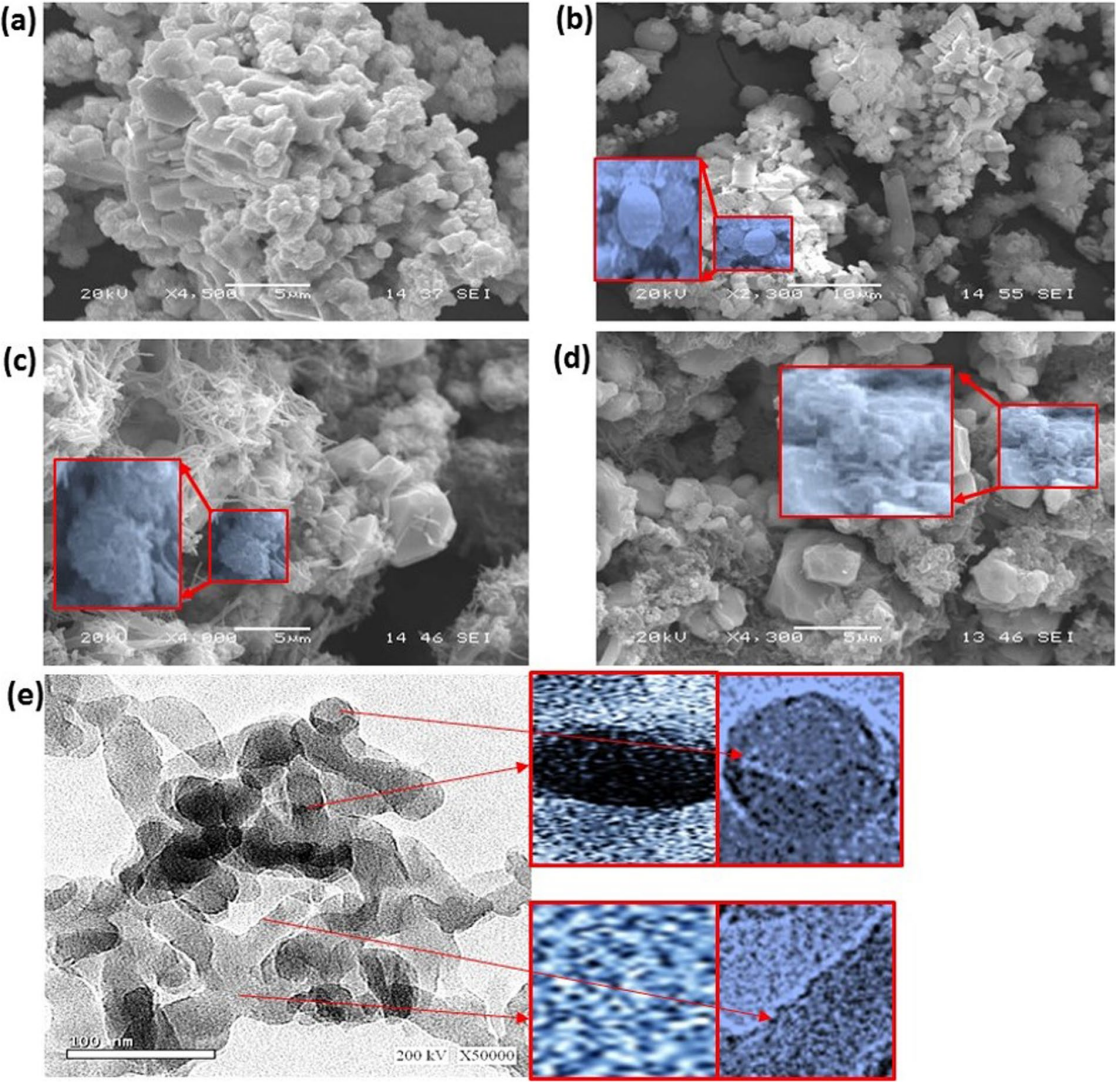
SEM analyses of (**a**) PbS nanoparticles and (**b–d**) PANI/PbS core-shell nano/microcomposites (I, II, and III). (**e**) HR-TEM of PANI/PbS core-shell nano/microcomposite (II).

The morphology of the PbS nanoparticles is investigated by SEM analyses as shown in Fig. 3(a). This image clearly illustrates the formation of spherical and ribbed nanoparticles with an average particle size of 100 nm. Moreover, SEM for PANI/PbS core-shell nano/microcomposites (I, II, and III) are shown in Fig. 3(b to d), respectively, From the figure, the formation of nano/microcomposites with spherical, ribbed, and fiber shapes are formed with average particles sizes of 250 nm. Moreover, with increasing of the aniline concentration during the preparation of core-shell nano/microcomposites, the fibers shapes increase as shown in the insets of Fig. 3(b–d).

The TEM analysis of PANI/PbS core-shell nano/microcomposite (II) is shown in Fig. 3(e). From the figure, the PbS nanoparticles (black color) are embedded in PANI (gray color) indicates the formation of PANI/PbS core-shell nano/microcomposite. The spherical and ribbed shapes of PbS nanoparticles appear clearly in the magnified parts of Fig. 3(e) (top insets). In addition, the nanoporous nature of PANI shell appears in the magnified parts of Fig. 3(e) (bottom insets). The estimated shell thickness is about 15–35 nm.

**Figure 4:**
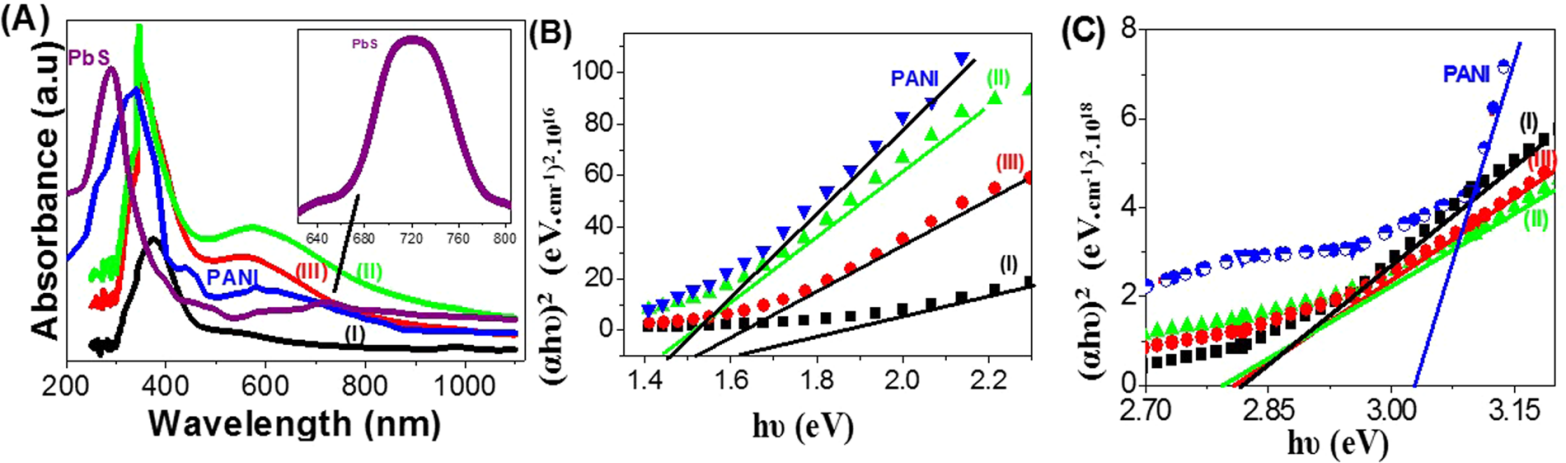
(**A**) Optical absorbance analyses and (**B**,**C**) band-gap values of PbS and PANI nano/microparticles, and PANI/PbS core-shell nano/microcomposites (I, II, and III).

The study of optical properties of the PANI and PbS nano/microparticles, and PANI/PbS core-shell nano/microcomposites (I, II, and III) is one of the essential factors to extend their applications. The optical absorbance spectra PANI, PbS nano/microparticles and the composites with different aniline concentration values are represented in Fig. 4(A).

In the case of PANI nano/microparticles (green line), the spectrum shows strong absorption peak centered at 333 nm in the UV-region, and two broad peaks at 439 and 600 nm in the visible region. The sharp peak is due to Π-Π* transitions from the benzenoid ring^43^, whereas the two broad peaks in the visible region are due to high conjugation of the aromatic polymer chain^44^. While, in the case of PbS nanoparticles, the spectrum shows the presence of a sharp peak at UV region at ~290 nm and another small broad one at 720 nm near-IR region (inset of Fig. 4(A)) attributed to intrinsic band-gap absorption of PbS and structural defect absorption, respectively^45^.

In addition, in the case of PANI/PbS core-shell nano/microcomposites (I, II, and III), the spectra show strong absorption peak centered at 360 nm in the UV-region and another broad peak at 570 nm in the visible region. From the figure, the formation of PANI/PbS nano/microcomposites makes an enhancement of the absorbance values especially in the UV and visible regions. This absorbance increases with the increase of the aniline concentrations in the composites from 0.01 to 0.03 M (composite (II)), then decrease with further increasing of aniline concentration to 0.05 M.

Based on direct allowed transition type the optical band gap of powder samples can be estimated using Tauc’s equation (Eq. 1) ^46^.

1 $$\alpha ={(hv-{E}_{g})}^{1/2}/hv$$

where 𝛼 is the absorption coefficient, A is the absorbance of the sample, *E*_*g*_ is the optical band gap, h is the Planck constant, and *v* is reciprocal of the wavelength. *α* is given by Eq. (2)^47,48^.

2 $$\alpha =2.303\,x\,{10}^{3}A\beta /lC$$

where β is the density of PANI (1.36 g/cm^3^) and PANI/PbS core-shell nano/microcomposites (3.2 to 3.6 g/cm^3^), *l* = 1.0 cm = the path of the quartz cell, and C is the concentration of the powder in the suspension.

The band gaps values for PANI nano/microparticles and PANI/PbS core-shell nano/microcomposites (I, II, and III) with different aniline concentrations are estimated by extrapolation of the straight line of the plot of (αhυ)^2^ versus hυ, as shown in Fig. 4(B,C). PANI has two band gaps at 1.45 and 3.03 eV; this is matched with the previous study of Shaban *et al*.^47^. Also, each nano/microcomposite has two band gaps. The first band gaps values, Fig. 4(B), are 1.62, 1.41, and 1.52 eV for nano/microcomposites (I, II, and III), respectively. In which the values of band gaps decrease with increasing of the aniline concentrations in the nano/microcomposites from 0.01 to 0.03 M, then increase with the further increase of the aniline concentration to 0.05 M. In addition, the second band gaps are represented in Fig. 4(C). From the figure, the band gaps values have the same behavior of the first values, in which they decrease with the increase of the aniline concentrations in the nano/microcomposites (I, II, and III) and have values of 2.82, 2.79, and 2.81 eV, respectively. Moreover, the band gap of PbS nanocrystallites is calculated from Fig. S1 (Supplementary data). This figure shows two band gap values at 1.93 and 3.11 eV corresponding to the two absorption band that observed in the optical spectrum of PbS, Fig. 4(A). From the obtained band gap values of PbS, PANI, and PANI/PbS core-shell nano/microcomposites (I, II, and III), the enhancement in the optical properties of the composites is due to the significant effect of the synergistic interaction of PbS and PANI matrix^49^, this is confirmed by the FT-IR shift peaks and X-ray analyses. Moreover, the values of the band gaps clearly refer to the enhancement of the optical properties of the nano/microcomposites (I, II, and III), in which nano/microcomposite (II) has the optimum values that qualify it for application in H_2_ generation systems.

### Photoelectrochemical H_2_ generation

**Figure 5:**
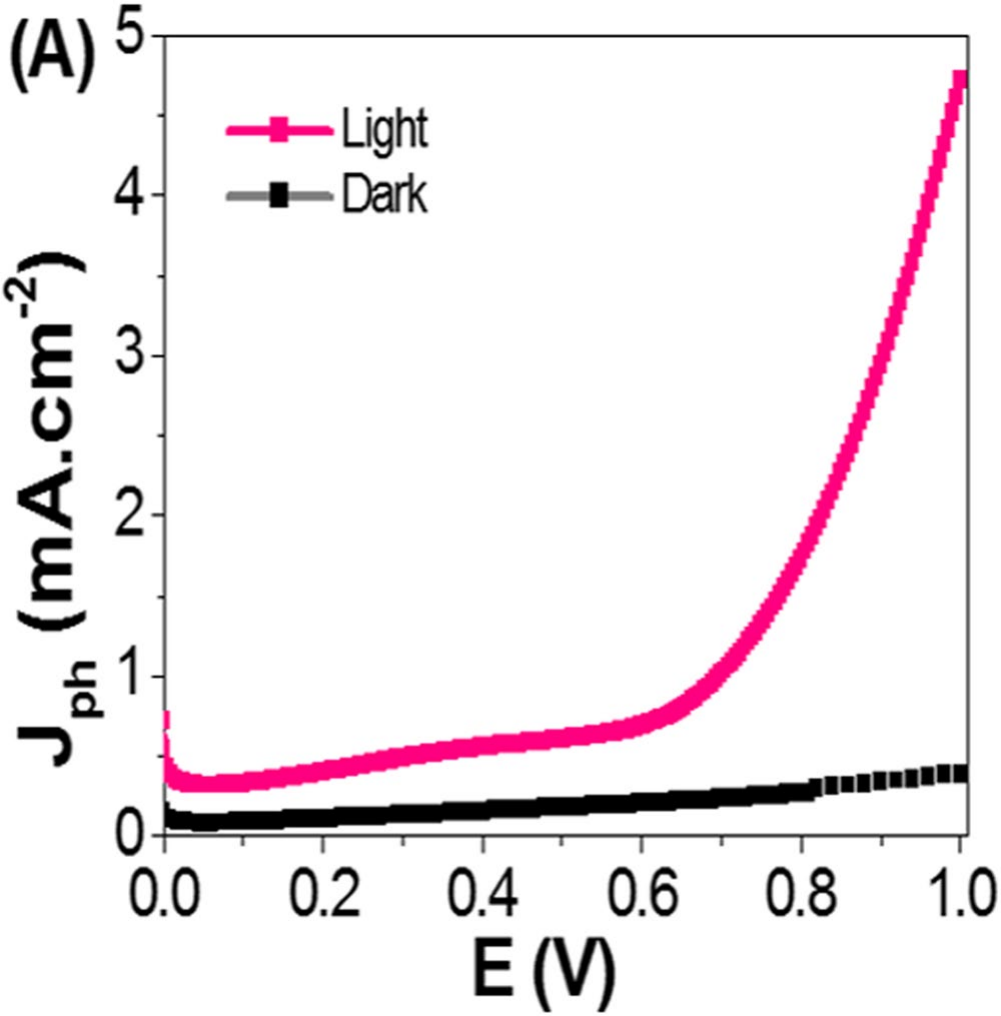
Photocurrent-voltage curves of PANI/PbS/ITO membrane electrode under illumination of 400 W metal-halide Lamp (**A**) without the optical filter in dark and light and (**B**) with optical filters of different wavelengths.

The photoelectrochemical (PEC) behaviors of the PANI/PbS/ITO nanocomposite (II) membrane electrodes supported on ITO glass was measured in dark and light without optical filters as shown in Fig. 5. The PEC behaviors were measured under illumination of 400 W metal-halide Lamp in 100 ml of 0.3 M Na_2_S_2_O_3_ solution at room temperature (25 °C) with a sweep rate of 1 mV/s. The prepared electrode with 1 cm^2^ surface area is used as the photoanode, and Pt-electrode of the same area is used as the counter electrode. Upon exposure to light, the large surface area of the nano/microcomposite electrode will produce a high density of electron-hole pairs, which will motivate the splitting of H_2_O molecules under the effect of light to carry out the hydrogen generation reaction.

From Fig. 5, the current density-voltage (J_ph_-E) behaviors of the PANI/PbS/ITO electrode is strongly affected by light exposure. The current densities are 0.4 and 4.76 mA.cm^−2^ in dark and light, respectively. In addition, the photocatalytic behavior of the nano/microcomposite membrane electrode is improved and the current density increased by increasing the applied voltage. Also, the values of the current density of the electrodes are firmly affected by the supporting ITO material that acts as a current collector.

PbS nanostructure acts as the main photocatalyst material in the PANI/PbS/ITO electrode for H_2_ generation process, from which most of the photoelectrons produced^50^. To study the effect of PANI on the activity of PANI/PbS/ITO electrode for H_2_ generation process, the photocurrent density-voltage (J_ph_-E) curves of PANI/ITO electrode in the dark and artificial light illumination are measured and presented in Fig. 2S. From the figure, PANI/ITO electrode is slightly affected by light and acts as a photocathode, in which the values of the J_ph_ in dark and light at −1 V are −0.061 and −0.066 mA.cm^−2^, respectively. PANI consists of mobile free electrons responsible for conductivity with asymmetry nature. Matveeva *et al*.^51^ discussed the charge transfer behavior of PANI/ITO interface. The protonation of the ITO surface introduces some sort of charge exchange sites or current passes that reduce the additional barrier for charge transfer processes on the ITO/PANI interface and make easier charge transfer through them.

Figure 3S shows the variation of J_ph_ with the electric potential of the electrodes configurations under the illumination of monochromatic light. The optical filters of different wavelengths from 390 to 636 nm are used to control the wavelength of the illumination at 25 °C. From this figure, the J_ph_ values decrease with increasing of the wavelengths from 390 to 490 nm, with further increasing of the wavelengths, there are small changes in the values of J_ph_. Then, the maximum J_ph_ values are obtained at wavelengths of 390 nm, which matched with the absorption peak positions (Fig. 4(A). Also, the distinct behavior of the photoanodes can be tentatively attributed to the enhanced solar absorption by the PANI/PbS core-shell nano/microcomposite that can cover a significant portion of the solar spectrum.

**Figure 6:**
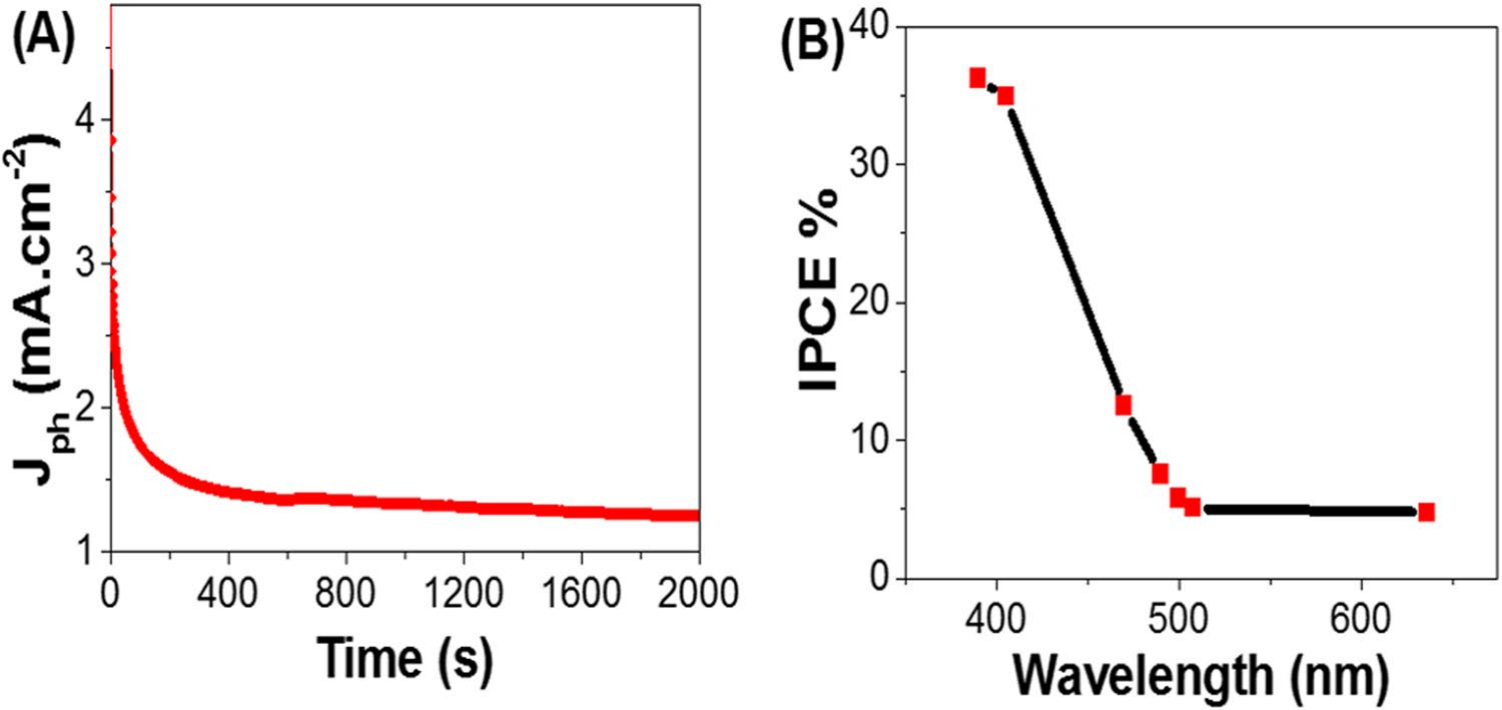
(**A**) current – time characteristic under illumination of 400 W metal-halide Lamp and (**B**) IPCE efficiency as a function of wavelength.

The stability of the PANI/PbS/ITO nano/microcomposite (II) membrane electrode is investigated for a prolonged time and shown in Fig. 6. During these experiments, a small bias voltage of 0.75 V is applied between the photoanode and the counter electrode to overcome any external losses of the measuring system. From Fig. 6, the J_ph_ values are decreased sharply from 4.76 to 1.4 mA.cm^−2^ as the time increased to 400 s. By further increasing the time to 2000 s, J_ph_ values almost remains constant due to the increasing accumulation of the ionic charges, which suggests a longer lifetime of the PANI/PbS/ITO nano/microcomposite electrode. Also, with increasing the time to 2000 s, the high density of surface states may lead to a significant pinning of the Fermi level that can facilitate the participation of these defect states in the surface oxidation process, leading to small degradation of the nano/microcomposite electrode^52^.

The enhanced IPEC properties of the PANI/PbS/ITO nano/microcomposite membrane electrode is further confirmed by measuring the incident photon-to-current conversion efficiency (IPCE) under monochromatic illumination conditions as shown in Fig. 6(B). Such analytical measurements can also give a meaningful insight into the contribution of PANI/PbS nano/microcomposite in the conversion of the incident photons into charge carriers. The IPCE is determined at an applied potential of 1 V from Eq. (3) ^53^:

3 $${\rm{IPCE}}( \% )=1240.\frac{{{\rm{J}}}_{{\rm{ph}}}}{{\rm{\lambda }}\,.{\rm{\rho }}}.100$$

where λ is the wavelength of the illuminating monochromatic photons and ρ is the illuminating light power density (mW.cm^−2^). From Fig. 6(B), Based on the optical behavior of the nanocomposite, the optimum values for IPCE % are obtained at 390 and 405 nm with values of 36.5 and 35.2%, respectively. With increasing of the wavelengths, the IPCE % values decrease till reach 5% at 500 nm.

**Figure 7:**
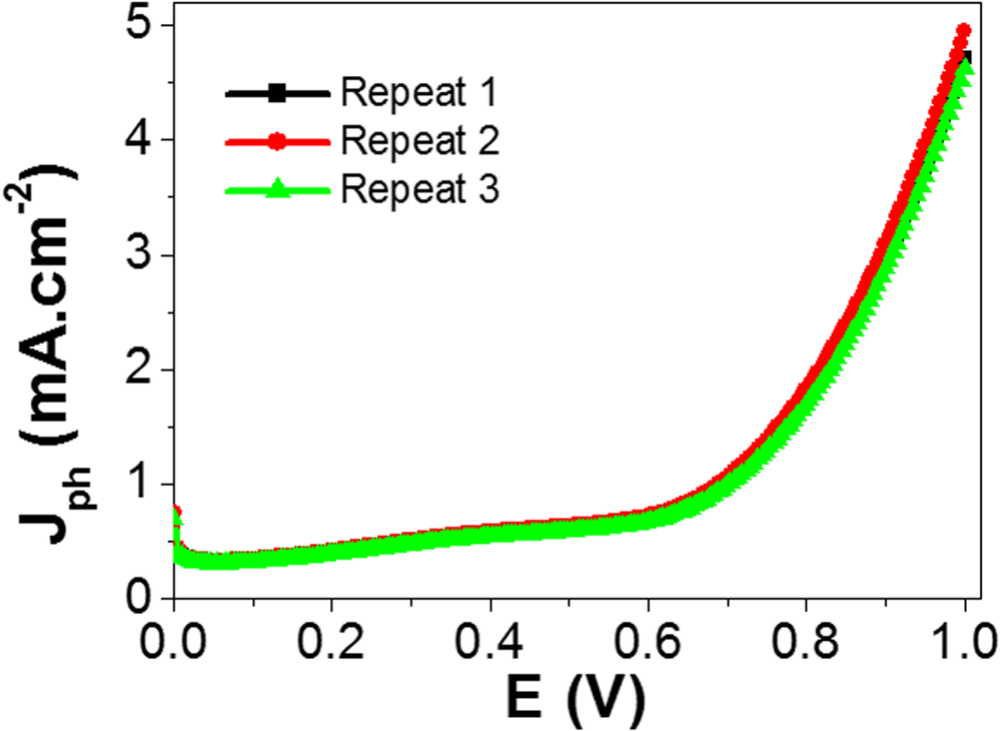
Reproducible studies of photocurrent density-voltage curves of PANI/PbS/ITO membrane electrodes under illumination of 400 W metal-halide Lamp without the optical filter.

Form the experimental point of view; the reproducibility study is very important for confirming the obtained data^54^. Figure 7 shows the J_ph_ – E curves of the PANI/PbS/ITO electrode under illumination of 400 W metal-halide lamp without the optical filter for three repeats or cycles. Then, the statistical analyses are carried out depending on the reproducible studies of J_ph_ – E curves. From Fig. 7, the J_ph_ values for the PANI/PbS/ITO electrode are measured three times with Relative Standard Deviation (RSD) of 6.1% and a mean value of 4.78 mA.cm^−2^.

**Figure 8:**
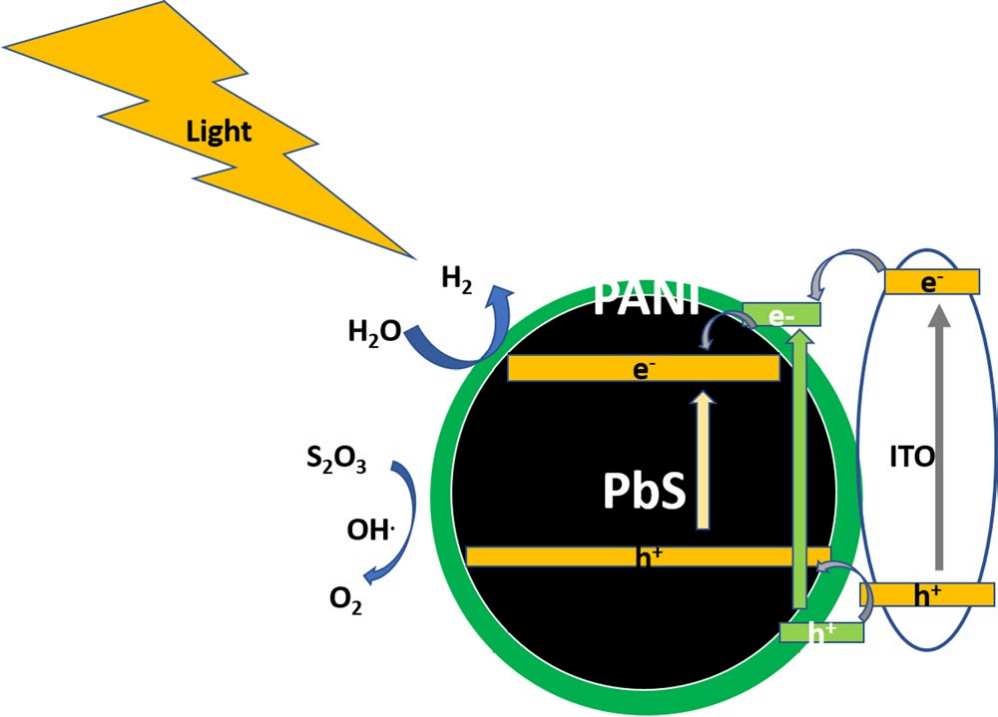
Mechanism of photochemical H_2_ generation from H_2_O using core-shell nano/microcomposite membrane electrode, PANI/PbS/ITO.

Based on the previous experimental results for H_2_ evolution using 0.3 M Na_2_SO_3_ solution as a sacrificing agent, the reaction mechanism is proposed in Fig. 8 for a better understanding of the photocatalytic process of PANI/PbS/ITO membrane photoanode. The surface electrons on ITO are transferred to the surface of the PANI nano/microparticles. Moreover, under the effect of artificial light, the levels of PANI are split, in which the electrons excitations take place. The transfer of electrons occurs from HOMO to LUMO levels^55^. Because of the existence of the potential difference between PANI and PbS levels, the LUMO electrons of PANI injected to the conducting band (CB) of PbS nanoparticle. Then, the PbS nanoparticles serve as electron donor material, in which the excited electrons can transfer from the core to out of the shell to H_2_O because of electron tunneling process^56^. In the other side, the photogenerated holes on HOMO of PANI can migrate to PbS VB level. The Na_2_S_2_O_3_ (sacrificing agent) accept the holes from PbS for the O_2_ evolution with the help of OH^.^ Radicals^55^. This electron-hole transition process is repeated with the lifetime of the prepared nanocomposite electrode that indicates a photocatalytic activity of the photocatalyst powder^57^.

## Conclusion

PbS nanoparticles were prepared by chemical bath deposition method at 80 °C, while, PANI nano/microparticles were prepared by the oxidative polymerization process. PANI/PbS core-shell nano/microcomposites (I, II, and III) were prepared by chemical polymerization of aniline in the presence of (0.05 g) PbS nanoparticles using aniline concentrations of 0.01, 0.03, and 0.05 M, respectively. The different concentrations of aniline were dissolved in 0.0005 M H_2_C_2_O_4_ at 298 K. The chemical structures and morphologies of the prepared nano/micromaterials were determined using FT-IR, X-ray, SEM, and HR-TEM analyses. In addition, the optical absorbance analyses and bandgap calculations were carried out for all prepared nano/micromaterials. The core-shell nano/microcomposite (II) has the optimum optical data with strong absorption peak centered at 360 nm in the UV-region and another broad peak at 570 nm in the visible region. Moreover, it has the optimum bandgaps values of 1.41 and 2.79 eV. The core-shell nano/microcomposite (II) membrane electrode supported on ITO glass was applied as photoanode for H_2_ electrogeneration from H_2_O in the presence of Na_2_S_2_O_3_. The H_2_ electrogeneration took place using I-V characteristic behavior in the presence and absence of optical filters of different wavelengths at 298 K. Finally, IPCE under monochromatic illumination conditions were calculated. The optimum values for IPCE % were 36.5 and 35.2% that obtained at 390 and 405 nm, respectively.

## Experimental Procedures

### Preparation of PbS nanoparticles

The preparation process of PbS nanoparticles occurred by the chemical bath deposition method at 80 °C using the precursors Pb(NO_3_)_2_, thiourea, NaOH and Triethanolamine (C_6_H_15_NO_3_) (Oxford Laboratory, India, 99%). In 100 ml beaker, 0.05 M Pb(NO_3_)_2_ (Oxford Laboratory, India, 99%), 0.04 M triethanolamine (C_6_H_15_NO_3_) (Oxford Laboratory, India, 99%) and 0.2 M NaOH (El Nasr pharmaceutical company, Egypt, 99%) were dissolved well using the ultrasonic device for 30 min. The mixture then stirred at 80 °C for 15 min. After that, 0.06 M thiourea (Alpha chemical company, India, 99.1%) was added, and the mixture was further stirred for 15 min at the same temperature. The black precipitate was formed indicated the formation of PbS particles. Finally, the PbS particles were washed several times using warm distilled water and dried at 60 °C for 12 h.

### Preparation of PANI nano/microparticles

PANI nano/microparticles were prepared by a sudden oxidative polymerization method. 0.03 M aniline (Rankem company, India, 99%) was dissolved in 0.0005 M H_2_C_2_O_4_ (El Nasr pharmaceutical company, Egypt, 99%) under the effect of ultrasonic waves. By the same approach, 0.06 M (NH_4_)_2_S_2_O_8_ (Winlab company, UK, 98.5%) was dissolved well in 0.0005 M H_2_C_2_O_4_. Moreover, the dissolved (NH_4_)_2_S_2_O_8_ was added over the dissolved aniline suddenly. In addition, the green precipitate was formed indication the polymerization of aniline to PANI (emeraldine salt). Then, the PANI was washed several times with warm water and dried at 60 °C for 12 h.

### Preparation of PANI/PbS core-shell nano/microcomposites (I, II, and III)

The prepared PbS nanoparticles were used as cores for the deposition of PANI shells by the oxidative polymerization process of aniline to form PANI/PbS core-shell nano/microcomposites. The oxidative polymerization was carried out using three different aniline concentrations; 0.01, 0.03, and 0.05 M, in which the nano/microcomposites are labeled as (I, II, and III), respectively. Aniline was dissolved in 20 ml (0.0005 M) H_2_C_2_O_4_ at 298 K and then mixed with 0.05 g PbS nanoparticles. The polymerization process occurred using three different concentrations of (NH_4_)_2_S_2_O_8_ as an oxidant, such that the concentrations of oxidant to aniline in all solutions are 2:1. The three solutions were put on ultrasonic for 1 h. After that, the samples were stirred for 5 h at 298 K using the magnetic stirrer device. The dark green precipitates (emeraldine salt) are formed indicating the completion of the polymerization process and formation of PANI/PbS core-shell nano/microcomposites (I, II, and III). Finally, the three nano/microcomposites (I, II, and III) are washed well with distilled H_2_O and dried at 60 °C for 12 h.

### Preparation of PANI/PbS core-shell nano/microcomposite (II) membrane electrode supported on ITO glass

PANI/PbS core-shell nano/microcomposite membrane electrode supported on ITO glass (Aldrich, 20 Ω) was prepared using the optimized optical analyses and applied for the photoelectrochemical H_2_ generation. 3% of the nano/microcomposite (II) was mixed with 3% Dibutyl phthalate (DBP) (Middle-east company, Egypt, 99.5%) and 3% polyvinyl chloride (PVC) (Middle-east company, Egypt, 99.5%). All the components were mixed well and dissolved in minimum volume of Tetrahydrofuran (THF) (Middle-east company, Egypt, 99.5%). The resulting mixture was transferred into a Petri dish of 5 cm diameter. The total weight of constituents in each batch was fixed at 0.35 g. The Petri dish was then covered with a filter paper and left to dry in air. To obtain a uniform electrode thickness, the amount of THF was kept constant, and its evaporation was fixed for 24 h. The thickness of the electrode is ~0.2 mm. 10 mm diameter disk was cut out from the prepared electrode and glued to one side of ITO glass slide using Ag-THF paste.

### Nano/microparticles characterization

The crystal structure of the deposited nano/microstructures PANI, PbS, and PANI/PbS were studied using high-resolution X-ray diffractometer system (model: PANalyticalX’Pert Pro, Holland) with CuK ${\rm{\alpha }}$ radiation (${\rm{\lambda }}$ = 1.5406 A°), operated at 45 kV and 40 mA. The XRD patterns were recorded in the 2θ range 10–90°. The pattern was analyzed by matching the observed peaks with the standard patterns provided by JCPDS files. Also, scanning analyses were carried out using Scanning Electron Microscope, SEM, (Model: ZEISS SUPRA 55 VP and ZEISS LEO, Gemini Column), and Transmission Electron Microscope, TEM, (JEOL JEM-2100 TEM). In addition, Fourier Transform Infrared Spectroscopy (FTIR) measurements were carried out using Shimadzu FTIR-340 Jasco spectrophotometer. Finally, the optical absorption spectra of the prepared PANI, PbS, and PANI/PbS nano/micromaterials were measured using Shimadzu UV spectrophotometer (M160 PC) at room temperature in the range 200–1100 nm.

### Photoelectrochemical H_2_ generation test

The photocatalytic hydrogen electro-generation experiments were performed by PANI/PbS core-shell nano/microcomposite (II) membrane electrode supported on ITO glass. The photoelectrochemical I-V and I-T behaviors were measured using Keithley measurement – source unit (2400 SourceMeter, A Tektronix company). Nano/microcomposite (II) membrane electrodes (1 cm^2^) used as a working electrode, while Pt-electrode with the same dimensions was used as a counter electrode. 100 ml of 0.3 M Na_2_S_2_O_3_ (El Nasr pharmaceutical company, Egypt, 99%) was used as the source electrolyte. The cell was exposed to an artificial light lamp (blended metal halide lamp 400 W, China) provided with series of linear optical filters.

## Electronic supplementary material

Supplementary data
