# Supplementary material for: Preparation of polyaniline/PbS core-shell nano/microcomposite and its application for photocatalytic H2 electrogeneration from H2O
Source: Sci Rep. 2018 Jan 18;8:1107. doi: 10.1038/s41598-018-19326-w (PMC5773669; doi:10.1038/s41598-018-19326-w)
Supplement: Supplementary file 1 — Supplementary data [file 41598_2018_19326_MOESM1_ESM.pdf]

Supplementary information

Preparation of polyaniline/PbS core-shell nano/microcomposite and its application for photocatalytic H_2 electrogeneration from H_2O

Mohamed Rabia[1,2], H.S.H Mohamed[3], Mohamed Shaban[1]*, S. Taha[3]

[1]Nanophotonics and Applications Lab, Physics Department, Faculty of Science, Beni-Suef University, Beni-Suef 62514, Egypt.
[2]Polymer Research Laboratory, Chemistry Department, Faculty of Science, Beni-Suef University, Beni-Suef 62514, Egypt.
[3]Physics Department, Faculty of Science, Fayoum University, EL-Fayoum, Egypt.

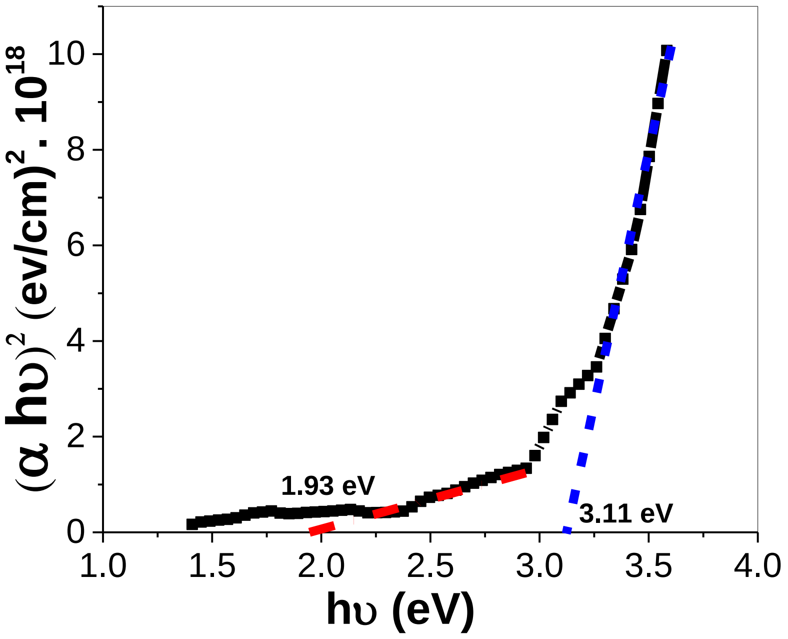

Figure S1. **The band gap values of PbS nanoparticles.**

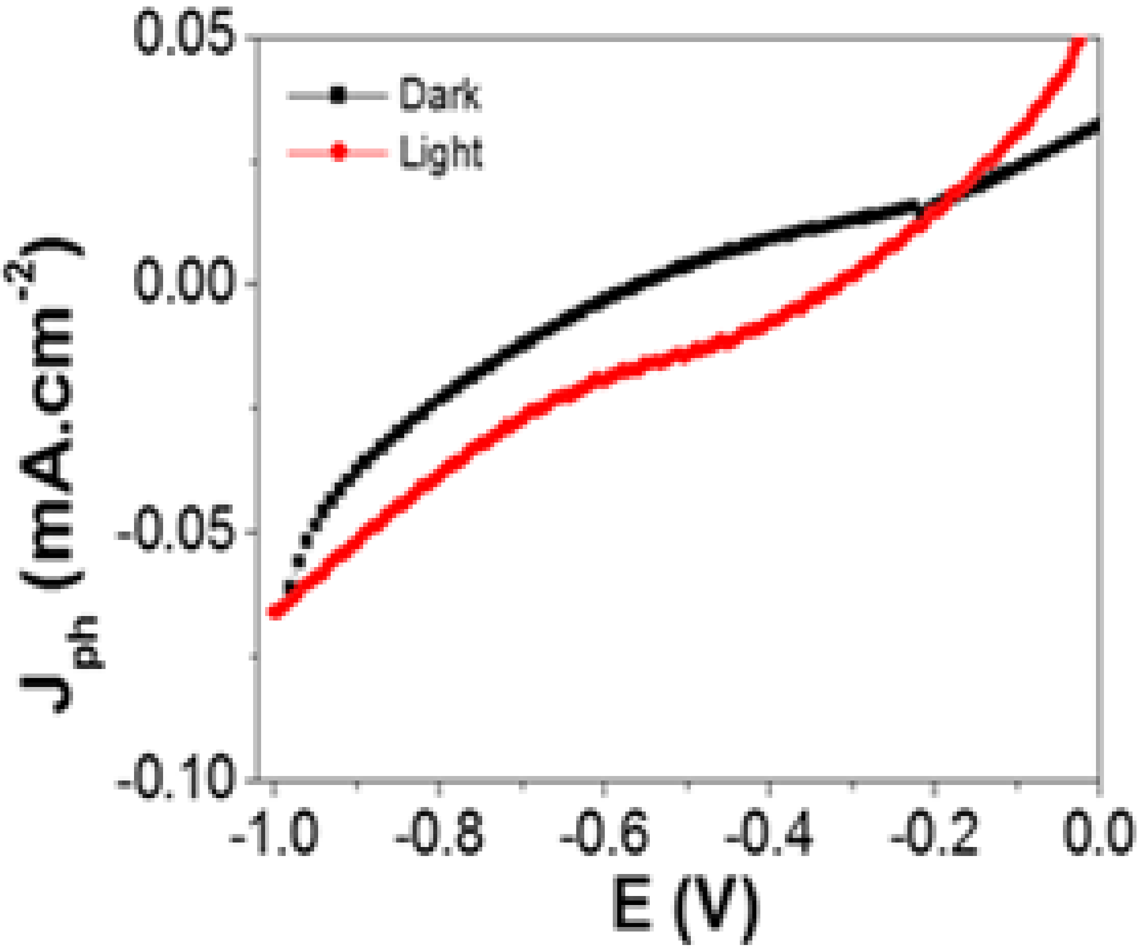

Figure S2. **Photocurrent density-voltage curves of PANI/ITO electrode in the dark and light under illumination of metal-halide Lamp without the optical filter.**

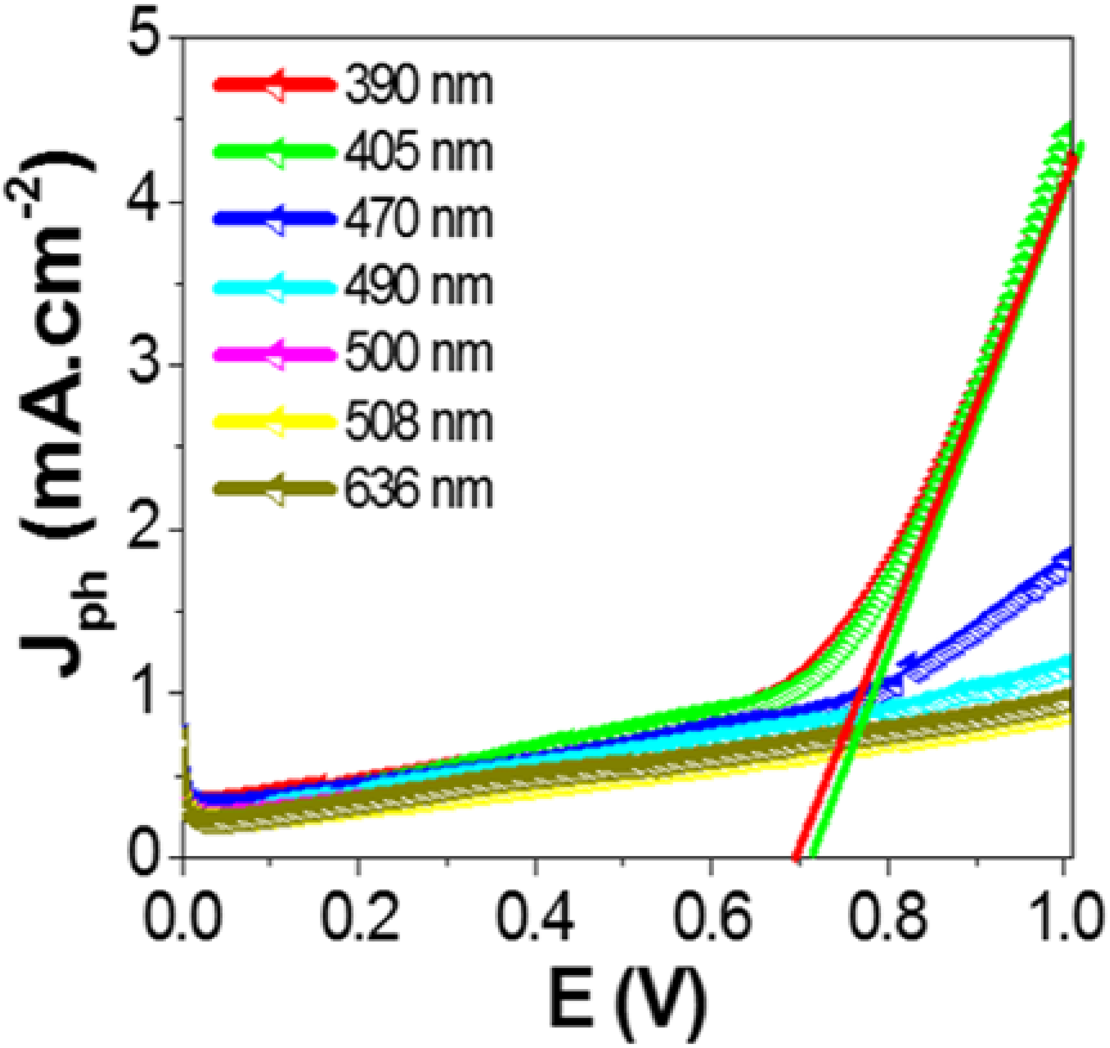

Figure S3. **Photocurrent-voltage curves of PANI/PbS/ITO membrane electrode under illumination of 400 W metal-halide Lamp with optical filters of different wavelengths.**
